# Supplementary material for: Content-rich biological network constructed by mining PubMed abstracts
Source: BMC Bioinformatics. 2004 Oct 8;5:147. doi: 10.1186/1471-2105-5-147 (PMC528731; doi:10.1186/1471-2105-5-147)
Supplement: Additional File 2 — The original results of the above study (non-essential files are deleted to keep the file size under the limit set by BMC bioinformatics). [file 1471-2105-5-147-S2.bz2 › chilibotAdditionalFile2/dip20/230ID7931156E749/html/UL5_UL8.html]

 


 **UL5** and **UL8** 
  
Found 42 abstracts in PubMed, retrieved 20.  
 

 What does Google say? 
 PDF only 
| .edu only 

---

**Interactive relationship** (e.g. stimulation, inhibition, etc)

**Neutral relationship**- The interactions of the UL52 protein with the  **UL8**  and  **UL5**  proteins were analysed by using the yeast two hybrid system.  Ref: 10501495 J Gen Virol, 1999
- Inhibition of herpes simplex virus replication by a 2 amino thiazole via interactions with the helicase component of the  **UL5**   **UL8**  UL52 complex.  Ref: 9696789 J Virol, 1998
- Surface plasmon resonance measurements demonstrated an interaction between ICP8 and the  **UL5**  52 8 heterotrimer but not with the  **UL5**  52 subassembly or the  **UL8**  protein alone.  Ref: 9278436 J Biol Chem, 1997
- These data suggest that  **UL8**  protein mediates an interaction between the  **UL5**  52 core enzyme and ICP8 that optimizes the utilization of ICP8 covered DNA templates during DNA replication.  Ref: 8702953 J Biol Chem, 1996
- On the other hand, the mutant protein retains its ability to interact with  **UL5**  as indicated by copurification and with  **UL8**  as indicated by a supershifted band in the gel mobility shift assay.  Ref: 10075707 J Biol Chem, 1999
- We demonstrate here that the  **UL8**  protein is required for the helicase activity of the  **UL5**  52 subassembly on long duplex DNA substrates >30 nucleotides with a single stranded DNA loading site fully coated with the virus encoded single strand DNA binding protein, ICP8.  Ref: 9278436 J Biol Chem, 1997
- Deletion mutants of the herpes simplex virus type 1  **UL8**  protein effect on DNA synthesis and ability to interact with and influence the intracellular localization of the  **UL5**  and UL52 proteins.  Ref: 9344911 Virology, 1997
- Moreover,  **UL8**  protein was required to reverse the inhibitory effect of ICP8 on the DNA dependent ATPase and primase activities of the  **UL5**  52 proteins.  Ref: 8702953 J Biol Chem, 1996
- We found that while ICP8 stimulated the DNA helicase activity of the  **UL5**  52 proteins up to 3 fold, maximum stimulation by ICP8 required the presence of  **UL8**  protein.  Ref: 8702953 J Biol Chem, 1996

**Non-interactive relationship** (e.g. studied together, co-existance, homology, etc.)

- The DNA helicase primase complex of HSV 1 consists of three sub units with molecular weights of 1 0 00 and 7 00, encoded by genes UL52,  **UL5**  and  **UL8** , respectively.  Ref: 9421876 Virus Genes, 1997
- Herpes simplex virus type 1 encodes a helicase primase complex composed of the products of the  **UL5** , UL52, and  **UL8**  genes.  Ref: 8662872 J Biol Chem, 1996
- Since this pattern can form in cells infected with mutants which are defective in  **UL5** ,  **UL8** , UL9, or UL52 in the presence of polymerase inhibitors C.  Ref: 9151871 J Virol, 1997
- The primase activity of the intertypic complex was 10 fold lower than that of HSV 1  **UL5**  HSV 1  **UL8**  HSV 1 UL52.  Ref: 9445019 J Virol, 1998
- Herpes simplex virus type 1 encodes a heterotrimeric helicase primase composed of the products of the  **UL5** , UL52, and  **UL8**  genes.  Ref: 9020191 J Biol Chem, 1997
- All three subunits of this complex  **UL5** ,  **UL8** , and UL52 are required for viral DNA replication in all cell types.  Ref: 9445019 J Virol, 1998
- We show that the HSV 1  **UL5** ,  **UL8** , UL29, UL30, UL42, and UL52 gene products along with the AAV Rep68 protein are sufficient to initiate replication on duplex DNA containing the AAV origins of replication, resulting in products several hundred nucleotides in length.  Ref: 11581393 J Virol, 2001
- Herpes simplex virus type 1 encodes a heterotrimeric helicase primase complex composed of the products of the  **UL5** , UL52, and  **UL8**  genes.  Ref: 11278618 J Biol Chem, 2001
- The herpes simplex virus type 1 DNA helicase primase is a heterotrimer encoded by the  **UL5** ,  **UL8** , and UL52 genes.  Ref: 8702953 J Biol Chem, 1996
- The intertypic complex HSV 2  **UL5**  HSV 1  **UL8**  HSV 1 UL52 was purified and biochemically characterized.  Ref: 9445019 J Virol, 1998
- The  **UL5** ,  **UL8** , and UL52 genes of herpes simplex virus type 1 encode a multisubunit assembly that possesses primase, DNA helicase, and DNA dependent nucleoside triphosphatase activities.  Ref: 9013584 J Biol Chem, 1997
- T157602 inhibited reversibly the helicase activity of the HSV  **UL5**   **UL8**  UL52  **UL5**  8 52 helicase primase complex with an IC50 concentration of compound that yields 50% inhibition of 5 microM.  Ref: 9696789 J Virol, 1998
- Herpes simplex virus type 1 expresses a heterotrimeric helicase primase, the subunits of which are encoded by the viral  **UL5** ,  **UL8**  and UL52 genes.  Ref: 10501495 J Gen Virol, 1999
- While the  **UL8**  component of the helicase primase is not required for enzymatic activities by a subassembly of the  **UL5**  and UL52 proteins, only the holoenzyme  **UL5**  8 52 was stimulated by ICP8.  Ref: 9129659 J Gen Virol, 1997
- The results shown in this paper are consistent with the model that the five proteins, ICP8,  **UL5** ,  **UL8** , UL9, and UL52, form a scaffold.  Ref: 12634381 J Virol, 2003
- The ordered assembly of the herpes simplex virus HSV type 1 replication apparatus leading to replication compartments likely involves the initial assembly of five viral replication proteins, ICP8, UL9, and the heterotrimeric helicase primase complex  **UL5**   **UL8**  UL52, into replication foci.  Ref: 12634381 J Virol, 2003
- The herpes simplex virus type 1  **UL5** ,  **UL8** , and UL52 genes encode an essential heterotrimeric DNA helicase primase that is responsible for concomitant DNA unwinding and primer synthesis at the viral DNA replication fork.  Ref: 9593724 J Biol Chem, 1998
- The assembly of complete HSV replication compartments and incorporation of BrdU were both abolished by treatment with phosphonoacetic acid and by omission of any one of the seven viral replication proteins,  **UL5** ,  **UL8** , UL9, UL42, UL52, SSB, and Pol, that are essential for viral DNA replication.  Ref: 9060678 J Virol, 1997
- The herpes simplex virus type 1 HSV 1 helicase primase, an essential component of the viral DNA replication machinery, is a trimeric complex of the virus coded  **UL5** ,  **UL8** , and UL52 proteins.  Ref: 9344911 Virology, 1997
- An assembly of the  **UL5**  and UL52 subunits retains both enzymic activities, and the  **UL8**  protein has been implicated in modulating these functions, facilitating efficient nuclear uptake of the complex and interacting with other viral DNA replication proteins.  Ref: 9344911 Virology, 1997
- The ability of the  **UL8**  mutants to facilitate efficient nuclear localization of UL52 in the presence of coexpressed  **UL5**  was examined by immunofluorescence.  Ref: 9344911 Virology, 1997
- To further characterize the function of the  **UL8**  subunit, we have examined its effect on the activities of the  **UL5**  52 core enzyme using DNA templates covered by the herpes simplex virus type 1 single strand DNA binding protein ICP8.  Ref: 8702953 J Biol Chem, 1996
- The UL6 locus of pseudorabies virus PRV was analyzed to reveal a gene cluster with homology to herpes simplex virus  **UL5** , UL6, UL7 and  **UL8** , Epstein Barr virus BBRF1 and BBRF2, and Kaposi sarcoma associated herpes virus ORF43 and ORF42.  Ref: 9458304 Oncol Rep< MedlineTA>Oncol Rep, 1996
- Herpes simplex virus type 1 encodes a heterotrimeric helicase primase complex that is composed of the products of the  **UL5** , UL52, and  **UL8**  genes.  Ref: 10075707 J Biol Chem, 1999
- The Herpes simplex virus type 1 primosome consists of three subunits that are the products of the  **UL5** ,  **UL8** , and UL52 genes.  Ref: 9278436 J Biol Chem, 1997
- the heterotrimeric helicase primase complex, encoded by  **UL5** ,  **UL8** , and UL52 genes, is believed to unwind duplex viral DNA at replication forks.  Ref: 11642367 Prog Nucleic Acid Res Mol Biol, 2001
